# Integration of Telehealth in Routine Perinatal Care: A Model of Care for Primary Healthcare Clinics in Saudi Arabia

**DOI:** 10.7759/cureus.47295

**Published:** 2023-10-18

**Authors:** Razaz Wali

**Affiliations:** 1 Primary Healthcare, Ministry of National Guard Health Affairs, Jeddah, SAU; 2 Family Medicine, King Abdullah International Medical Research Center, Jeddah, SAU; 3 Family Medicine, King Saud Bin Abdulaziz University for Health Sciences, Jeddah, SAU

**Keywords:** protocol, postnatal clinic, pregnancy counseling, virtual, model of care, saudi arabia, perinatal care, telehealth

## Abstract

This study aims to introduce a new model of antenatal/postnatal care that integrates virtual clinics with the current model of care, including a discussion on the current model, pre-existing barriers, and prenatal framework, and the need for transition to telehealth beyond the pandemic. In antenatal primary health care centers, such as King Abdulaziz Medical City (KAMC), low-risk antenatal/postnatal care receives clinical care through complete physical attendance in antenatal/postnatal clinics in primary care clinics for pregnancy follow-up and in tertiary hospitals for fetal ultrasound and invasive procedures if needed. Pregnancy is confirmed through a regular family medicine (FM) clinic where risk assessment through history, physical examination, and investigations are carried out. If the pregnant woman is at low risk, she will be started on folic acid, 1 mg or 5 mg based on the risk assessment (if it was not received before). Pregnant women will be given a telehealth appointment for the lab results. Concomitantly, the pregnant women will receive an appointment in the antenatal clinics, which board-certified family physicians run. High-risk patients will be referred to the hospital for further care. Current postnatal care is delivered through regular booking with the FM clinic through physical attendance sometimes, and virtual care is provided upon physican/patient request. Current care meets the past quality care and patient expectations. However, with the current Saudi Vision 2030 and after the experience with virtual care during the COVID-19 pandemic, the current services need to move a step forward to meet the rapidly developing medical care/needs in Saudi Arabia. Various challenges must be addressed, and new models must be included in clinical care for pregnant and postnatal women. Introducing virtual antenatal/postnatal care to the current care could be a new era in maternity primary health care; this model will move the clinical care provided to pregnant/postnatal women a step forward that meets the excellence of high-quality, evidence-based medical care.

## Introduction and background

Maternal health is considered a public health indicator globally. In Saudi Arabia, antenatal and postnatal care is essential; a healthcare worker attends to 99.4% of pregnancies [1]. The primary goal of prenatal care is to ensure the delivery of a healthy newborn, maintain good maternal health, and minimize complications. Many countries consider maternal mortality and morbidity a significant challenge. Half of maternal deaths occur in the perinatal period. Studies proved that appropriate use of healthcare resources, including maternity and postnatal care, can improve maternal and infant outcomes [2].

During the COVID-19 pandemic early in 2020 and 2021, healthcare institutions and primary healthcare centers were challenged to deliver health services, especially to vulnerable groups, including pregnant and postnatal women. COVID-19 is known for its high pathogenicity, effect on the respiratory system, and admission to intensive care units [3]. Most countries, including Saudi Arabia, implemented a curfew in which a person could not leave his/her home without official permission. During this pandemic, most outpatient services were transformed into telehealth, with care delivered through phone calls.

Telemedicine, telehealth, or mobile health can be used interchangeably, and they indicate the use of technology and its applications to deliver a health service. It can include virtual visits, phone calls, and mobile healthcare [4]. Since most of the population has mobile phones, this advantage can be used to deliver health services [5]. Women can receive antenatal or postnatal care via a phone or video call and learn how to self-monitor their pregnancy. Many perinatal services can be replaced by telehealth visits, such as asking about current health, fetal movement, health education and counseling, breastfeeding and screening for mental health, and even monitoring vital signs with a home blood pressure monitor. A study conducted in the USA reported that telehealth is a potentially convenient, ideal approach to delivering health services and can replace some physical visits. It also ensures continuity of care [6,7].

Advantages of maternity telehealth

The literature reports many advantages of maternity telehealth. A study measuring pregnant women's satisfaction with telehealth indicated that 86% of the women rated their experience as very good or good, and the women thought it could be a reasonable approach to delivering a healthcare service [8,9]. A systemic review in the USA showed that antenatal telehealth improved obstetric outcomes, breastfeeding rate, and early access to a high-risk pregnancy clinic [10]. Another randomized controlled trial that compared blended antenatal care with pure in-person care showed no difference in fetal or maternal outcomes in low-risk pregnancies, in addition to easy access and eliminating the attendance barriers [11,12]. An Australian study reported that a 50% reduction in personal antenatal visits did not compromise the outcome [13].

Maternity telehealth can improve access to care and reduce pregnancy stress without affecting the outcome with high personal and professional satisfaction and cost-effectiveness [8,14-16]. One study discussed that the quality of provided visits is more important for patient satisfaction than the number of visits itself [17]. In a cross-sectional study that surveyed women in the postpartum unit regarding their preference for the current antenatal and postnatal care, the women were willing to have diverse care methods, including the introduction of phone health and spacing of visits as an alternative way of care delivery [18].

Maternity telehealth beyond the COVID-19 pandemic

After the successful implementation of telehealth in maternity services in many healthcare institutes, Sullivan and his group recommended that tele-maternity care should continue beyond the pandemic due to the favorable outcome, and it can be a replacement for some maternity care visits [19,20]. It can reduce unnecessary visits and infection risk and promote equality of health distribution and better utilization of resources, including less time and a lower workforce and delivery of care to a higher number of women to improve the quality of services.

Current existing antenatal/postnatal care system

According to the World Health Organization (WHO) guideline, a low-risk pregnant woman needs at least four visits at 12, 20, 28, and 36 weeks of gestation with the same safety outcome; these visits can be increased if needed [21]. Some countries still recommend more frequent visits depending on the risk factors, where pregnant women have their first contact in the first 12 weeks of gestation, with subsequent contacts taking place at 20, 26, 30, 34, 36, 38, and 40 weeks of pregnancy.

The antenatal care for a low-risk pregnancy in the primary healthcare centers in King Abdulaziz Medical City (KAMC) follows the shared care protocol with the tertiary center. This protocol was adapted from the American College of Obstetricians and Gynecologists (ACOG) guidelines, which offer 12-14 visits. It recommends a visit every month until 28 weeks and then every two weeks until the 36th week, when she will be referred to the hospital for weekly follow-up until delivery [22]. All high-risk pregnant women are referred to the obstetrics department for further management at the initial screening (high-risk pregnancy can be defined as any pregnancy that puts the mother or her baby at a health problem compared to a typical pregnancy).

The initial visits can predict many pregnancies’ adverse outcomes by integrating a complete history, physical examination, and laboratory tests. During the initial visit, a thorough history and comprehensive physical examination, including vital signs, are performed, in addition to a risk assessment. The initial antenatal laboratory tests and nuchal translucency (NT) ultrasound will be arranged, and folic acid will be prescribed. Many recommendations suggest the importance of the initial visits and recommend shifting the pyramid to initial visits rather than the third trimester [23]. In subsequent visits, vital signs, protein in urine, fundal height, and fetal heart are checked. In addition, there is a discussion about nutrition, exercise, pregnancy care, breastfeeding, and contraception plans. Many studies suggest that these visits can be replaced by telehealth [6].

Postnatal telehealth visits

Many pregnancy complications can arise during the postpartum period. An initial postnatal visit can be conducted through telehealth, where the entire history, breastfeeding assessment, physical examination, and laboratory tests are ordered; a follow-up physical visit can be performed between six and eight weeks to discuss contraception and reassess the risk factors. Globally, the attendance of women for postpartum care is low. Studies have proven that telehealth could improve access to postpartum care, especially in the first week after delivery [18,24].

One study by Arias and his group showed that telehealth improved screening for postpartum depression and the postpartum visit attendance rate but adversely affected the timely use of long-term contraceptions [25]. KAMC and its related primary healthcare centers in the Western region do not have an official postnatal clinic. Women in their postpartum period visit primary healthcare for medical reasons.

## Review

A model of integrating virtual care into antenatal/postnatal clinics

Healthcare centers in other counties developed protocols to incorporate perinatal telehealth in their services, primarily in outpatient clinics with obstetricians. However, no protocols were developed for the primary healthcare centers in the community setting to deliver professional telehealth care services.

Regarding the introduction of telemedicine to maternity care, some centers suggested eight onsite visits with an obstetrician and six online visits with a midwife or registered nurse; this system showed improved satisfaction with no difference in the fetal or maternal outcome [26]. Other guidelines suggested four in-person visits, one fetal ultrasound, and four virtual visits; these guidelines demonstrated similar outcomes [27,28]. However, a study by Carter et al. showed that low-risk pregnancy with more than 10 visits did not improve the pregnancy outcome or maternal satisfaction [29]. Antenatal care should be initialized based on the risk assessment, shifting the resources to high-risk pregnancies. This will ensure proper and effective use of resources and implementation of equity.

The ACOG fully supports the introduction of telehealth in low-risk maternity care and encourages physicians to adopt the technology in their practice [30]. To date, no guideline officially incorporates obstetric telehealth in routine antenatal and postnatal care, more specifically in antenatal care in family medicine (FM) practice. The ACOG recommends creating a guideline locally, and online antenatal care can be an option for low-risk pregnant or postnatal women [31]. Studies have shown that online antenatal services have many advantages, and adopting such programs is necessary in the era of Saudi Vision 2030 [32].

This protocol aims to use telecommunication and technology to deliver structurally organized, safe, high-quality, evidence-based patient care through technology and physician experience in the mother and her baby's best interest to provide safe, culturally acceptable healthcare for women in the prenatal period.

Objectives

This model aims to introduce a new model of antenatal and postnatal care that integrates virtual clinics with the current model of care to facilitate the uptake of preventive measures, decrease the defaulter rate, detect risks in a timely manner, reduce complications, and consult with patients in hard-to-reach areas or conflict settings within the scope of the Ministry of National Guard Health Affairs (MNG-HA) standards.

Purpose

Low-risk pregnant or postnatal women should receive clinical care visits through telehealth and telemedicine antenatal care programs to deliver preventive, promotive, and rehabilitative health services and optimize maternal health care. Maternity telehealth can improve early referral of high-risk pregnancies to secondary care while maintaining maternal satisfaction and better utilization of resources for low-risk pregnancies without compromising the fetal or maternal outcome.

This protocol can reduce the number of unnecessary visits, reduce the cost of the services, and empower mothers to take the lead in caring about their health through educational applications, courses, consultation, and rapid access visits. It is expected to provide a structure for future antenatal care that integrates telehealth in routine low-risk antenatal and postnatal care without risking fetal or maternal outcomes, safety, and satisfaction.

Description

**Figure 1:**
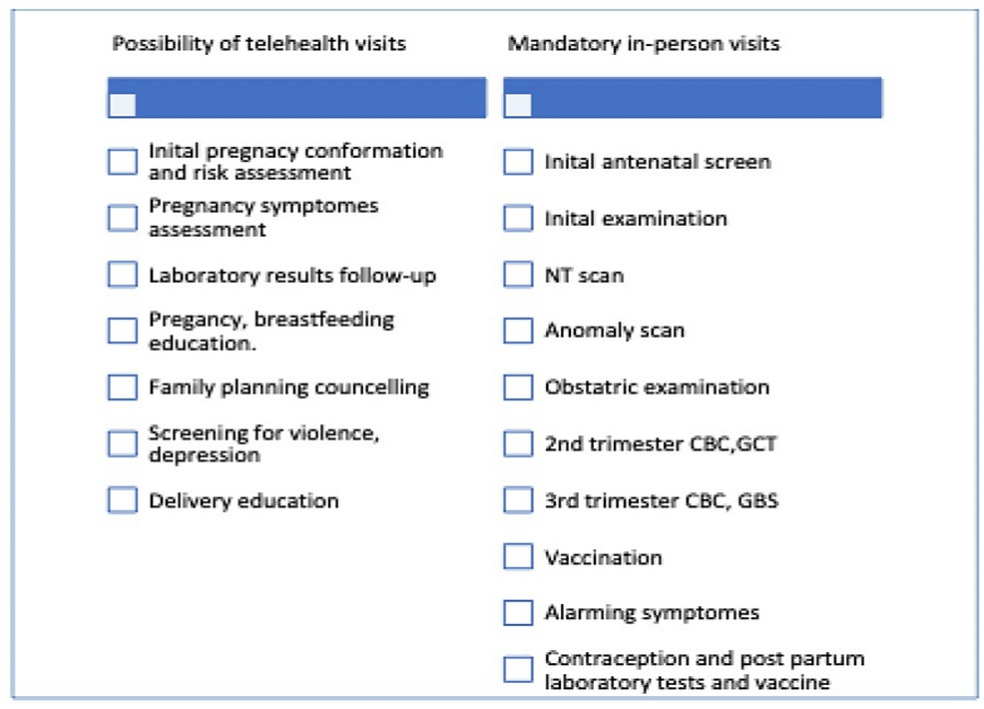
Antenatal and postnatal care elements that can be delivered through telehealth versus in-person visits. NT scan: nuchal translucency scan; CBC: complete blood count; GCT: glucose challenge test; GBS: group B *Streptococcus*

Many services can be provided through telehealth, including pregnancy, postnatal, and exercise education, delivery instructions, partner violence screening, blood pressure monitoring, blood sugar monitoring, mental health education, and follow-up (Figure 1). To implement this protocol, the needs should be addressed in structure, including the space, staffing, technology, incorporation of maternity health services in the existing electronic medical records, an appointment system, and a clear plan for high-risk patients. The process should consider the ethical aspects of the care, staff training, and communication with departments regarding concerns, including logistics, health promotion, and the quality department. Finally, it should be able to monitor the outcome regarding client and physician satisfaction, fetal and maternal outcome, comparison of physical versus telehealth cost-effectiveness, feasibility, and quality of care.

Suggested model of antenatal care

**Figure 2:**
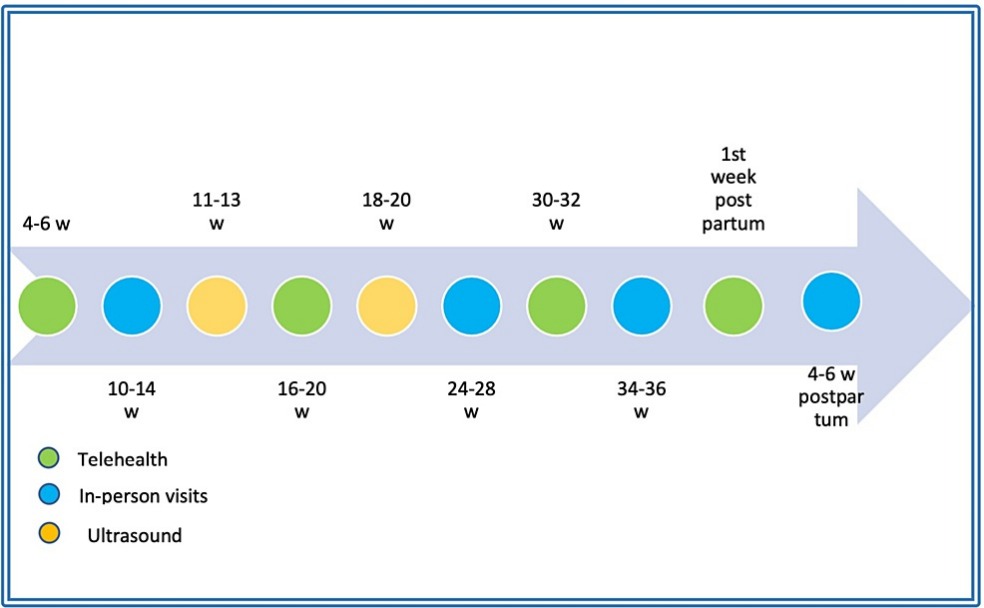
Suggested antenatal and postnatal visit timeline W: weeks

After a two-year implementation of telehealth services in the primary care setting during the COVID-19 pandemic and after the positive maternal satisfaction survey conducted in the primary health care [33], the literature was reviewed to develop a model that can be implemented in a primary healthcare setting and integrated into the shared care protocol in the MNG Hospital to accommodate the integration of telehealth in the routine antenatal and postnatal care and meet the objectives of the Saudi Vision 2030. This clinical protocol is informed by evidence from the literature to cover the three main aspects of care: risk assessment, preventive services, and therapeutic interventions (Figure 2, Tables 1, 2).

**Table 1:** Maternity telehealth team members and inclusion and exclusion criteria FM: family medicine; US: ultrasound; GP: general practitioner

Team	Board-certified FMs, midwifes, registered nurses, breastfeeding educators, patient educators, patients' services
Inclusion criteria	Low-risk pregnant women, initial booking visit, recent standard US, or visits with GP obstetricians
Exclusion criteria	High-risk pregnant women, symptoms of complications, medical or surgical history, abnormal laboratory, or US results

**Table 2:** Suggested protocol for in-person and telehealth antenatal or postnatal visit schedule Note: Pregnant or postnatal women can book a visit to family medicine clinics to discuss any concerns (continues easy access). Continuous risk assessment with each visit and the frequency of in-person visits can be changed accordingly. Patients can be offered optionally to buy a blood pressure cuff or fetal doppler or visit a nearby pharmacy or primary care center to measure their vital signs. W: weeks; ANC: antenatal clinic; OPD: outpatient department; PHC: primary healthcare; FM: family medicine; US: ultrasound; MFMU: maternal-fetal unit; CBC: complete blood count; GBS: Group B *Streptococcus*; GTT: glucose challenge test; TFT: thyroid function test

Weeks of pregnancy	Type of visit (location)	Delivered care	Manpower	Laboratory and referrals
Pre-pregnancy	Telehealth	Risk assessment, folic acid	Family physician	Consider referral for high risk, vaccination
4-6 w	Telehealth	Diagnosis and risk assessment, booking labs, folic acid, GP clinic	Family physician/staff physician	Pregnancy test, ANC workup, OPD in ANC
10-14 w	In-person (PHC)	Booking, risk assessment, fetal viability, discussion of laboratory results	Board-certified FM	Pregnancy education, breastfeeding education
11-13 w	In-person (hospital)	Dating scan, NT scan, radiology	US technician	US, MFMU
16-20 w	Telehealth	Current complaint, fetal movement, perinatal supplements	Board-certified FM, registered nurse, midwife	Pregnancy education, breastfeeding education
18-20 w	In-person (hospital)	Anomaly scan, radiology	US technician	US, MFMU
24-28 w	In-person (PHC)	Vital signs, obstetric examination, perinatal supplements	Board-certified FM, registered nurse, midwife	Laboratory work (CBC, GCT), anti D if needed, vaccination
30-32 w	Telehealth	Current complaint, fetal movement, perinatal supplements	Board-certified FM, registered nurse, midwife	Pregnancy education, breastfeeding education
34-36 w	In-person (PHC)	Vital signs, obstetric examination, perinatal supplements	Board-certified FM, registered nurse, midwife	Laboratory work (CBC, GBS), referral contraception discussion
First week postpartum	Telehealth	Postnatal checkup, screen for mental health, prenatal vitamins, childcare	Board-certified FM, registered nurse, midwife	CBC, TFT, 75 g GTT if GDM, breastfeeding education, contraception discussion
4-6 w postpartum	In-person (PHC)	Screen for mental health, perinatal supplements, child care contraception	Board-certified FM	Breastfeeding education, contraception discussion, vaccination

Stages of Implementation

The implementation stages are as follows: (1) preparing the foundation, infrastructure, safety netting, and program endorsement to the concerned departments; (2) appointment system (both physical and virtual); (3) electronic medical records; (4) virtual clinic platform and IT involvement; and (5) staff training.

Protocol Development Integrating Virtual Clinics in Routine Antenatal and Postnatal Care

This protocol will include four physical visits and four virtual clinics to the antenatal clinic, two postpartum visits, plus any appointments in a routine FM or emergency clinic (Table 2).

Protocol Approval

This protocol was reviewed by a consultant from the Obstetric Department, a consultant from the feto-maternal unit, and a senior FM consultant, and the approval from the primary healthcare director.

Implementation Stage

This protocol will be implemented in one primary healthcare for a pilot period.

Monitoring and Evaluation (Rapid Cycle Evaluation Approach)

This includes a continuous and frequent assessment of each process to test each domain (infrastructure, staff, and clients) regarding feasibility, acceptability, and access to care.

Limitations

Most studies measuring satisfaction were done during the COVID-19 pandemic lockdown, where the risk of getting infected was high and most clients preferred virtual visits. This protocol is implemented in the MNG-HA setting; generalization to other PHC settings might be different.

Recommendations

This protocol needs to facilitate the availability of Internet services to all pregnant and postnatal women and the development of online applications or programs to allow pregnant women to measure their blood pressure, urine analysis results, and blood glucose in the case of GDM. Additional studies are required to ensure the safety and quality of the provided health services in primary healthcare settings by using a case-control study to compare in-person visits versus integrated maternity care. After a couple of years, a follow-up study is needed to reassess various aspects, such as response rate, satisfaction, and, most importantly, the health outcome for both mothers and their babies.

## Conclusions

The COVID-19 pandemic provided many healthcare opportunities and challenges that healthcare should embrace to improve the existing healthcare systems. Although telehealth or telemedicine is a feasible and effective way to deliver health services to low-risk pregnant or postnatal women, it cannot replace physical attendance at clinics. Virtual clinic uptake and feasibility were tested during the COVID-19 pandemic and showed promising results, which can be reintroduced as a part of future antenatal care in Saudi Arabia.
